# Supplementary figures and images for: Community Risk Factors in the COVID-19 Incidence and Mortality in Catalonia (Spain). A Population-Based Study
Source: Int J Environ Res Public Health. 2021 Apr 4;18(7):3768. doi: 10.3390/ijerph18073768 (PMC8038505; doi:10.3390/ijerph18073768)

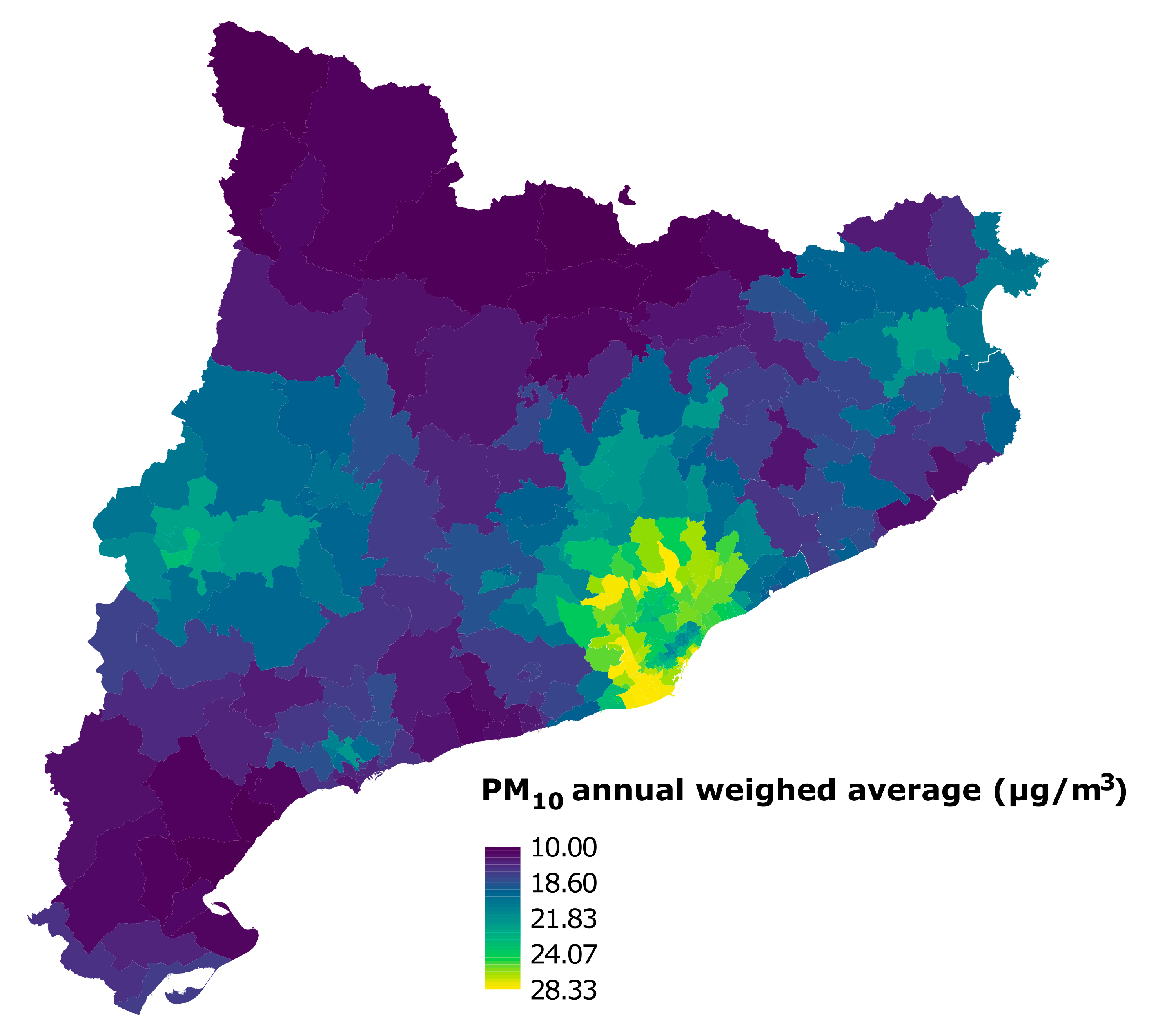

Supplement: Supplementary file 1 [file ijerph-18-03768-s001.zip › IMG_01/PM10_annual_weighed_average_2016.jpg]

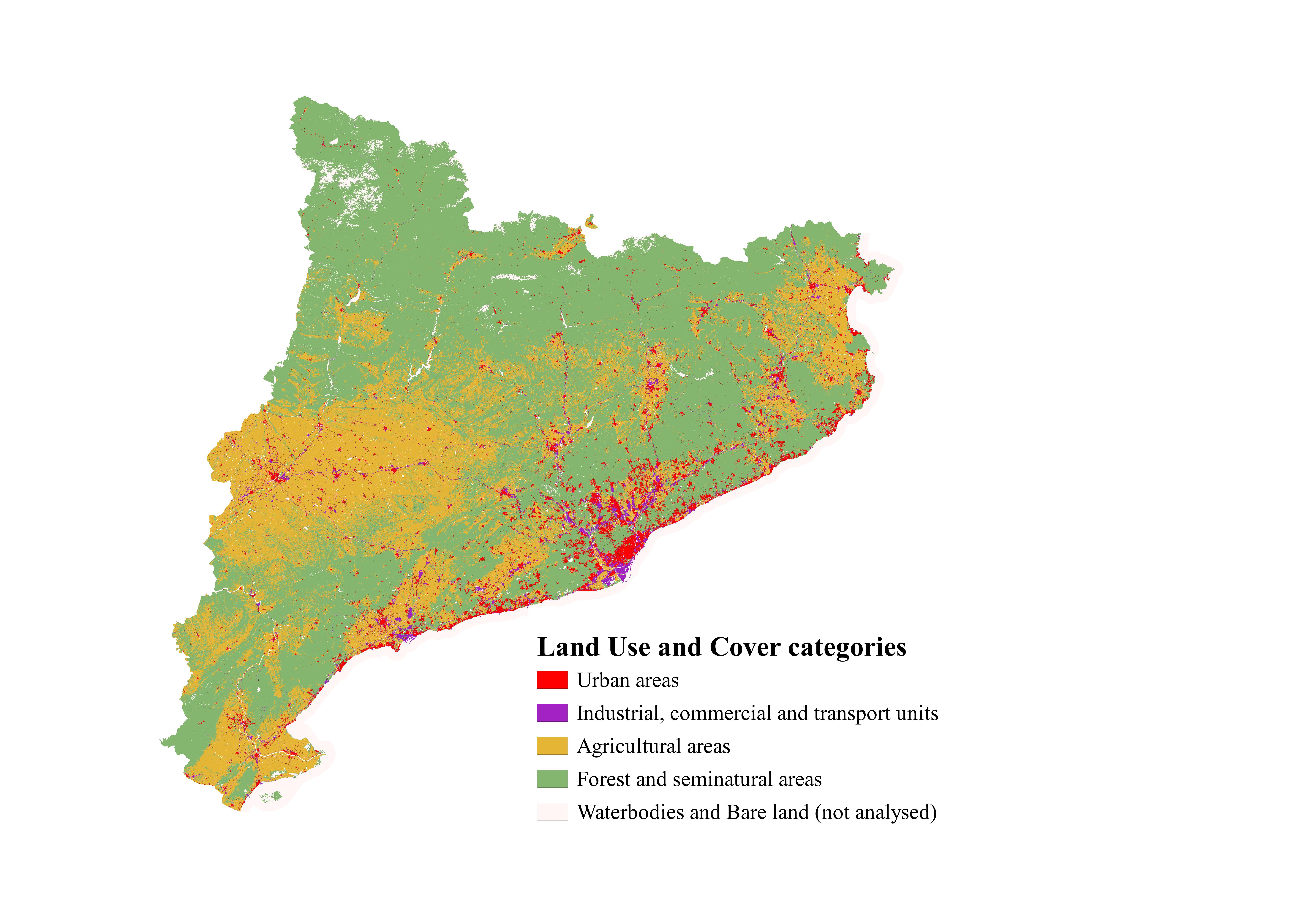

Supplement: Supplementary file 1 [file ijerph-18-03768-s001.zip › IMG_01/LULC_2.jpg]

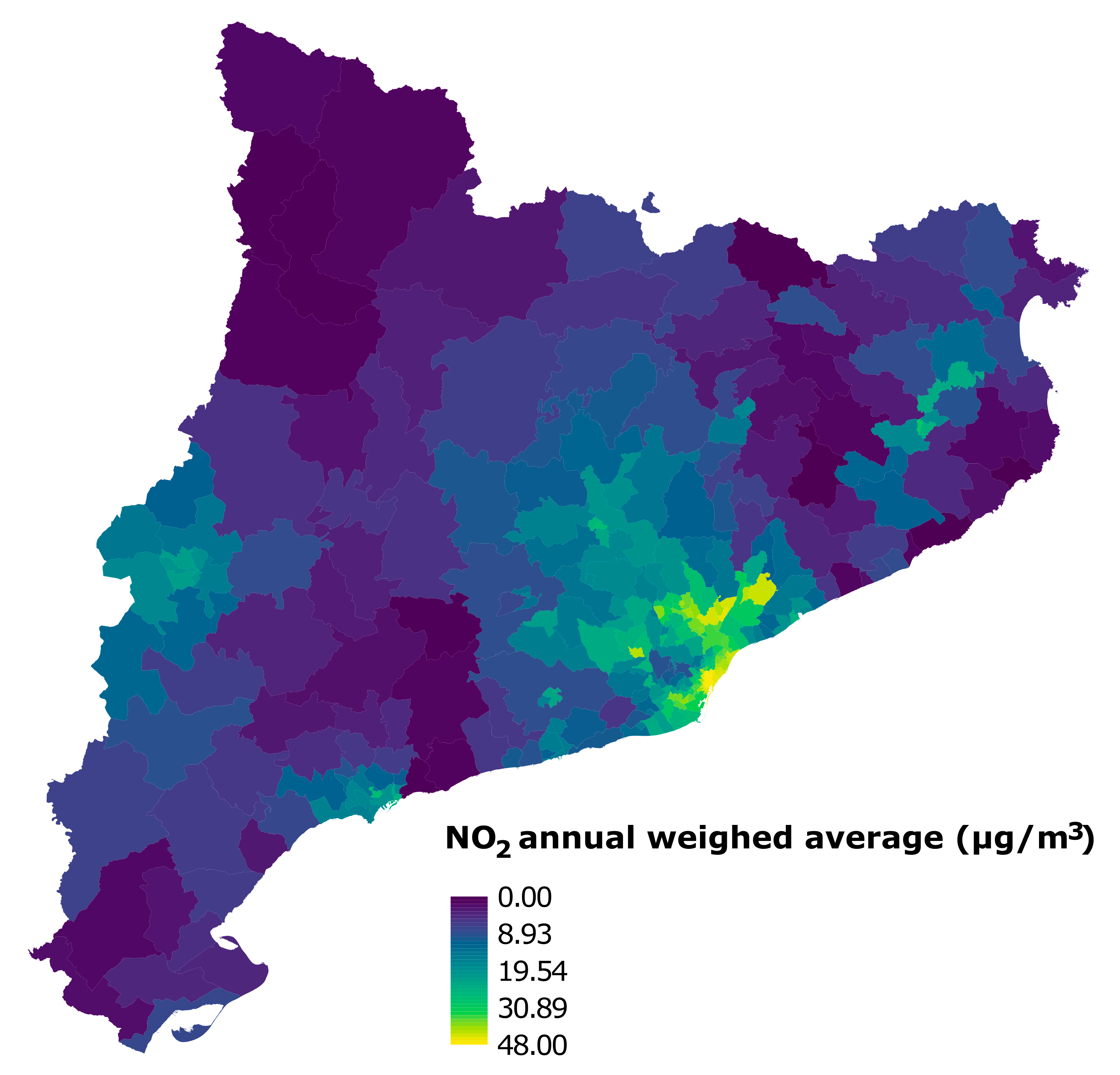

Supplement: Supplementary file 1 [file ijerph-18-03768-s001.zip › IMG_01/NO2_annual_weighed_average_2016.jpg]

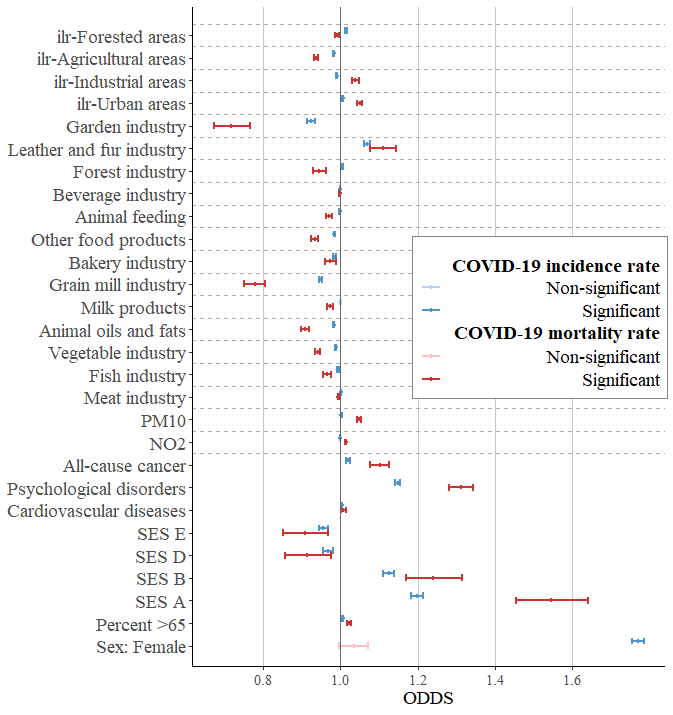

Supplement: Supplementary file 1 [file ijerph-18-03768-s001.zip › IMG_01/Incidence_mortality_CI_plot.png]
